# The Immediate Effect of Exercising in a Virtual Reality Treadmill (C-Mill) on Skin Temperature of a Man with Lower Limb Amputation

**DOI:** 10.1155/2023/7081000

**Published:** 2023-01-14

**Authors:** Fábio Marcon Alfieri, Caren da Silva Dias, Daniela Mitiyo Odagiri Utiyama, Denise Vianna Machado Ayres, Linamara Rizzo Battistella

**Affiliations:** ^1^Instituto de Medicina Fisica e Reabilitacao, Hospital das Clinicas HCFMUSP, Faculdade de Medicina, Universidade de Sao Paulo, Sao Paulo, SP, Brazil; ^2^Master Course in Health Promotion-Adventist University of Sao Paulo (UNASP), Sao Paulo, Brazil; ^3^Faculdade de Medicina FMUSP, Universidade de Sao Paulo, Sao Paulo, SP, Brazil

## Abstract

**Background:**

In amputees, exercising can impact the distribution of body temperature. The aim of this case report is to verify the acute effect of exercising in C-Mill on the temperature distribution in the lower limbs of a man with unilateral transfemoral amputation.

**Materials and Methods:**

The thigh and legs of a man with left distal transfemoral amputation were evaluated by thermography (infrared sensor FLIR T650sc) before and after a single 30-minute exercise session performed in a virtual reality treadmill device (C-Mill).

**Results:**

The thermographic evaluation showed a difference in temperature between the thighs both before and after the intervention. However, there was a decrease in asymmetry, which went from 4.0°C to 3.1°C in the anterior view and from 5.3°C to 2.9°C in the posterior view, after the intervention.

**Conclusion:**

Thermography allowed us to assess the difference in temperature in the lower limbs. Even though the temperature discrepancy has decreased after the single exercise session using the C-Mill, this difference persisted.

## 1. Introduction

It is known that rehabilitation provides functioning gains for people living with amputations. Especially in the case of lower limb amputations, mobility limitations may be a life-long rehabilitation challenge [1].

For this, reestablishing independent and functional gait is one of the main rehabilitation objectives. Rehabilitation technology such as virtual reality, video game-assisted therapy, and biofeedback systems can improve the rehabilitation of these individuals [1]. The C-Mill (ForceLink, Culemborg, Netherlands) consists of a 3-meter-long treadmill associated with body weight suspension and virtual reality, developed to practice gait adaptability. The C-Mill allows gait training to be associated with visual interaction. For example, the individual needs to step on or dodge targets that are displayed on the treadmill or on the screen in front of him/her while walking.

Visual projections help simulate walk by mimicking the real life, enabling individuals to adjust gait movements while interacting with obstacles and other tasks that demand both static and dynamic balance. The unweighting system provided by body weight suspension provides additional safety in case individuals loose balance during training [2].

The C-Mill is already reported to be useful to improve gait in patients with stroke [2]. Another study [3] reported that the C-Mill offers walking adaptability for patients with difficulties in gait and balance and that the training with this equipment is feasible and acceptable.

van de Venis et al. add that the treadmill and the screen provide a safe environment. The authors report that feasibility and efficacy have been achieved for treating people with neurological conditions such as stroke, ataxia, and multiple sclerosis [4].

Tests such as the Amputee Mobility Predictor Assessment Tool [5] are used to evaluate the success of therapy strategies. However, thermography is a completely painless, noninvasive, fast exam, without contraindications or collateral effects that can be used to objectively assess the distribution of body temperature and identify physiological dysfunctions [6, 7]. Once temperature is an indicator for many health conditions, it can be used to assess the vascular status in different body segments. Recent studies have used thermography as an assessment tool for individuals in rehabilitation treatment, such as for patients with stroke sequelae [8, 9]. A recent study carried out by our group found that there is a significant association between sensorimotor recovery and differences in cutaneous temperature and differences in tactile sensibility between both sides of the body in people with stroke sequelae [10].

Recently, our group published a case report that showed an improvement in the distribution of skin temperature in the lower limbs of a unilateral transfemoral amputee [11].

The aim of this case report was to verify the acute effect of exercising in the C-Mill on the temperature distribution in the lower limbs of a unilateral transfemoral amputee.

## 2. Case Report

The patient was a 67-year-old male with a right-sided, transfemoral amputation caused by a road traffic accident in June 2020. He weighed 71 kg, was 1.75 m, had a body mass index (BMI) of 28.8, and had been classified as a K3 level walker. He used two elbow crutches to move aroud. He had abilities or potential for ambulation with variable cadence. He could move around in the community independently overcoming most environmental barriers and had vocational, therapeutic, or exercise activities that demanded prosthetic use beyond simple locomotion [5].

**Figure 1:**
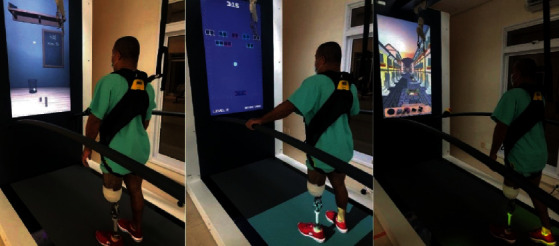
Exercise stages: stand, step, and walk.

In this case, the goal of rehabilitation was to resume an active lifestyle. Hence, the C-Mill exercise protocol used lasted for 30 minutes and included three different stages. During the first stage (stand), the patient remained standing and trained balance in this posture while tasks were projected in the front screen. The second stage (step) consisted of taking steps in different directions. The third stage (walk) demanded the patient to walk while simulating going around obstacles projected in the treadmill. Each modality lasted for 10, 5, and 15 minutes, respectively.  Figure 1 shows the patient during the three stages of the training.

Before and after this exercise protocol, the skin temperature was measured using infrared thermography in the same room where the C-Mill is located. The patient was instructed to stand up on a mark on the floor, 4 m away from the infrared camera and 0.4 m away from the wall. He remained standing in the same position during the image capture protocol.

The patient was instructed not to move his arms or legs unnecessarily or to scratch any region of the body before or during the procedure. He was also advised not to take hot baths or showers, use creams or powder, exercise, take in stimulants or beverages containing alcohol or caffeine, use nasal decongestants, smoke, or use vasoconstrictors or vasodilators two hours before the training session [12–15]. The temperature of the examination room was 21.4°C with 72% humidity.

Thermographic images were captured by a FLIR T650SC infrared sensor, with a resolution of 640 × 480 pixels, at a frequency of 30 Hz. This sensor can collect images with a temperature range from -40 to 70°C, with a precision of 1%, and a spectral band of 7.4-14 *μ*m, and noise equivalent temperature difference (NETD) < 20 mK. The examination used a thermic sensibility of 0.03°C with a colorimetric scale (color palette) considering a skin emissivity of 0.98.

**Figure 2:**
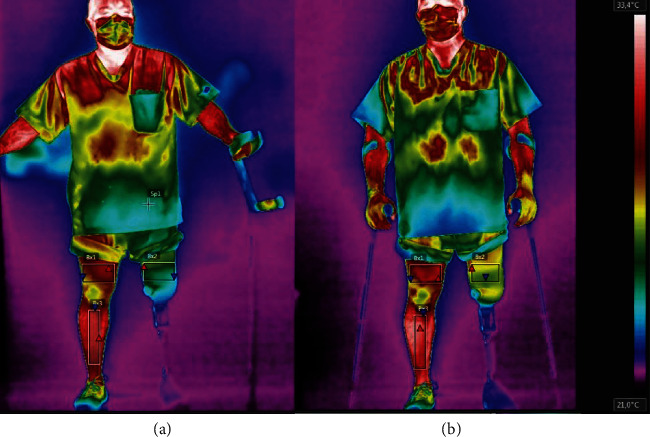
Anterior view of the patient before (a) and after (b) exercise.

**Figure 3:**
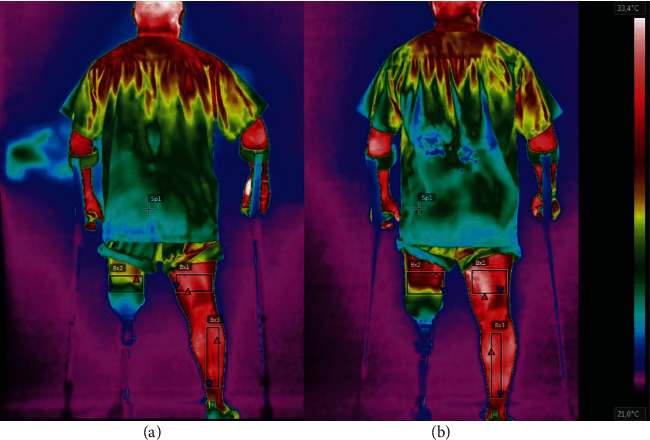
Posterior view of the patient before (a) and after (b) exercise.

The camera was standing by for image and temperature stabilization 15 minutes before data collection, being positioned perpendicularly to the patient. Thermograms were captured in both anterior and posterior views. The images were analysed with the FLIR Tools® software. Average temperature was measured in degrees Celsius (°C) at each region of interest (ROI). ROIs are rectangles determined by the following anatomical reference sites (Figures 2 and 3): thigh (5 cm above the superior border of the patella and the inguinal line) and leg (5 cm below the inferior border of the patella and 10 cm above the malleolus) [12, 13].

Table 1 shows the temperature of the patient's thighs and leg in both anterior and posterior views.

## 3. Discussion

After a single exercise session using the C-Mill, it was possible to observe a better distribution of skin temperature between the lower limbs of the volunteer. It should be noted here that the thermography exam can detect the temperature distribution between the body hemispheres and that this should be symmetrical. It means that the temperature in both sides of the body should be as similar as possible, accounting for some difference [6, 7, 12] (±0.3°C) in a thermoneutral environment [16–18]. Any significant asymmetry equal to or greater than 0.7°C can already be considered an abnormality and an indicator of some physiological or anatomical alteration [19].

Results show that the temperature of the right thigh in the anterior view remained practically the same before and after exercising and that in the posterior view it increased 0.4°C. However, the temperature of the left thigh increased 1°C in the anterior view and 2.8°C in the posterior view after the exercise session. Thus, after exercising, the difference between the sides diminished from 4.0° to 3.1° in the anterior view and from 5.3°C to 2.9°C in the posterior view. That is, a single exercise session, although not leaving the sides symmetrical [12], caused the difference between the sides to decrease.

Obviously, a single case and a single examination 30 minutes after the end of a single session do not allow the generalization of a possible improvement in the temperature distribution between the two sides of the body or its attribution to rehabilitation treatment. However, this result shows that thermography can be a useful tool for this type of functional assessment in which changes in body temperature distribution are present. A study carried out by our group has already shown that thermography could be useful for assessing stroke patients and that temperature distribution was more symmetrical soon after robot-assisted rehabilitation [20]. Another study also recently published by our group showed that the test is useful to identify patterns of temperature symmetry in a healthy individual and of asymmetry in an individual with vascular alteration, this being verified after a single session using active lower limb cycling exercises [21].

As with previous studies [2–4], this case report shows that the C-Mill can also be useful for amputees and that there were changes in temperature distribution between the body hemispheres in only 30 minutes. A possible hypothesis is that complete rehabilitation treatments using this and other devices could also bring improvements in functioning for these patients, as well as in the regulation of skin temperature between the sides of the body.

For this reason, future studies with using this type of rehabilitation intervention and evaluation, as well as others that assess functioning, are appropriate. Furthermore, a complete rehabilitation treatment may show if there is in fact an improvement and if there is a change in temperature distribution between the body hemispheres in amputees that lasts longer. Thus, the existence of a relationship between improving functional gait and the distribution of skin temperature may make thermography a recommended assessment strategy in amputee patients.

Finally, this case report shows that thermography can be used to assess skin temperature after amputation. Furthermore, after performing a single exercise session, a patient with transfemoral amputation showed a decrease in the discrepancy in skin temperature distribution between the two sides of the body.

## 4. Patient Perspective

The patient reported that the single exercise session performed on the C-Mill device was adequate and had no adverse effects and that the thermographic evaluation, as previously established in the literature, was quick and did not cause any discomfort, in addition to allowing quick visualization of its results.

## Figures and Tables

**Table 1:** Distribution of skin temperature before and after a single exercise session.

	Before	After
Anterior view		
Right thigh (°C)	29.1	29.2
Left thigh (°C)	25.1	26.1
Right leg (°C)	30.1	30.5
Posterior view		
Right thigh (°C)	30.9	31.3
Left thigh (°C)	25.6	28.4
Right leg (°C)	30.7	31.2

## Data Availability

The data from thermographic evaluations used to support the findings of this study are included within the article.
