# Corrigendum to “The Immediate Effect of Exercising in a Virtual Reality Treadmill (C-Mill) on Skin Temperature of a Man With Lower Limb Amputation”

**DOI:** 10.1155/crvm/9762585

**Published:** 2025-08-27

F. M. Alfieri, C. da Silva Dias, D. M. O. Utiyama, D. V. M. Ayres, and L. R. Battistella, “The Immediate Effect of Exercising in a Virtual Reality Treadmill (C-Mill) on Skin Temperature of a Man With Lower Limb Amputation,” *Case Reports in Vascular Medicine* 2023 (2023): 7081000, https://doi.org/10.1155/2023/7081000.

In the article titled “The Immediate Effect of Exercising in a Virtual Reality Treadmill (C-Mill) on Skin Temperature of a Man With Lower Limb Amputation,” an affiliation was omitted for the author “Linamara Rizzo Battistella” in error. The corrected author list and affiliation list should be as follows:

Fábio Marcon Alfieri^1,2^, Caren da Silva Dias^1^, Daniela Mitiyo Odagiri Utiyama^1^, Denise Vianna Machado Ayres^1^, Linamara Rizzo Battistella^1,3^

^1^Instituto de Medicina Fisica e Reabilitacao, Hospital das Clinicas HCFMUSP, Faculdade de Medicina, Universidade de Sao Paulo, Sao Paulo, São Paulo, Brazil

^2^Master Course in Health Promotion-Adventist University of Sao Paulo (UNASP), Sao Paulo, Brazil

^3^Faculdade de Medicina FMUSP, Universidade de Sao Paulo, Sao Paulo, São Paulo, Brazil

We apologize for this error.

